# Experiences of pregnant and parenting students at a university in Gauteng province

**DOI:** 10.4102/hsag.v29i0.2547

**Published:** 2024-04-22

**Authors:** Moroti E. Mahlangu, Tshiamo N. Ramalepa, Lucky O. Letswalo

**Affiliations:** 1 Department of Nursing Science, School of Healthcare Sciences, Sefako Makgatho Health Sciences University, Tshwane, South Africa

**Keywords:** pregnant students, parenting students, university, tertiary institution, experiences, pregnancy

## Abstract

**Background:**

Pregnancy and parenting in tertiary institutions is a worldwide concern. The number of pregnancies among tertiary students is increasing globally. About 16 million young women between the ages of 15 years and 19 years around the world became mothers and two million girls under the age of 15 years are reported to be pregnant every year. South African universities continue to report high rates of student pregnancies, and are looking for solutions to the crisis that female students are facing.

**Aim:**

The purpose of the study was to explore the experiences of pregnant and parenting students.

**Setting:**

At a university in Gauteng province, South Africa.

**Methods:**

A qualitative, exploratory and descriptive design was used in a study that was conducted at a university in Gauteng province, South Africa. Undergraduate pregnant and parenting students were sampled purposively, and the sample size was 15 participants. Semi-structured interviews were used to collect data and data were analysed using thematic analysis.

**Results:**

The findings of the study produced four themes, namely emotional experience during pregnancy, academic challenges during parenting, experiences during antenatal care, and students’ resilience during pregnancy and parenting.

**Conclusion:**

Pregnant and parenting students require emotional, academic and social support from the university and other stakeholders. The university should offer on-campus medical services such as antenatal care and provide academic support for pregnant and parenting students to help them achieve their academic objectives.

**Contribution:**

This study highlights the importance of developing support programmes that focus on pregnant and parenting students in universities.

## Introduction

Pregnancy and parenting at tertiary institutions is a worldwide concern. The number of pregnancies among tertiary students is increasing globally (Thabethe, Mulondo & Tugli 2020). Each year, about 16 million young women between the ages of 15 years and 19 years became mothers and two million girls under the age of 15 years report pregnancy worldwide (Jochim, Cluver & Meinck 2021). The majority of adolescent births are reported in sub-Saharan Africa, where school dropout is frequently observed (Jochim et al. 2021). Pregnant and parenting students are bona fide students who are pregnant at the time of registration at tertiary institutions or become pregnant during their studies and raise dependent children while facing academic expectations (Phiri, Nyamaruze & Akintola 2023). Pregnancy is the first step in the process of becoming a mother, and it is a time when all women experience significant physical and psychological changes. It is a time when many women spend money and energy preparing physically and emotionally for the birth of a child. Pregnancy impacts the mother’s life, especially in academic institutions where there is also a chance of financial and academic pressure (Phiri et al. 2023).

This period may be marked by high levels of physical and mental stress. The stressors may vary from physical tiredness, demanding academic work, decreasing funds, social isolation and depression. Young women who are pregnant in tertiary institutions have a variety of special pressures because of their age and social environment. Stressors including academic requirements, a lack of financial stability and relationship issues with their partners may have an impact on their mental health. Moreover, unexpected pregnancies endanger students’ psychological well-being at universities (Thabethe et al. 2020).

Universities have a role in supporting pregnant and parenting students because they are regarded as custodians in the absence of their parents. Younger pregnant and parenting students have greater health risks and poor education outcomes (Govender, Taylor & Naidoo 2020). According to a study by Malatjie et al. (2023), university administration and staff are ill-equipped with skills to assist pregnant and parenting students resume their academic studies after giving birth. Conversely, lecturers are sympathetic and comprehend the challenges faced by pregnant and parenting students (Malatji et al. 2023). Furthermore, other lecturers and students frequently criticise and humiliate pregnant and parenting students who exhibit resiliency by continuing their education while pregnant and after giving birth (Haufiku 2023).

In contrast to the aforesaid, a study conducted by Baney et al. (2022) highlighted instrumental support offered to adolescent mothers through practical assistance for pregnancy and parenthood. Educational institutions enabled them to continue their education and obtain their certificates and offered support such as being accommodating and understanding regarding the submission of assignments, offering a private area to express breast milk or feed their children, and also offering free on-site childcare for infants and toddlers (Baney et al. 2022). Pregnant and parenting students can only finish their studies when they have complete support from their significant others such as parents. Students who are pregnant or parenting can benefit from the assistance of their families and professionals including lecturers and healthcare experts (Jones et al. 2019). Emotional support is expected from healthcare professionals. It is also conceivable that healthcare professionals can be biased against pregnant and parenting students and not provide any support or empower them about the health information related to pregnancy, child care or parenting (Kessler et al. 2018). This suggests that social service providers, professional healthcare providers at campus health, and family members do not offer pregnant and parenting students enough informational support and also deprive them of learning about pregnancy and parenthood. Lastly, the lack of informational support among teenage mothers may suggest that healthcare and social service professionals need further instruction on the best practices for a more youth-centred approach (Decker et al. 2021).

Despite the high rate of student pregnancies across the world, governments vary widely in how much they use laws and policies to safeguard pregnant females’ access to education. In the United States of America (USA), Education Policy, Title IX, addresses methods for assisting students who are pregnant or nursing, including methods for administrators to meet their educational needs (Husch Blackwell 2021). This included pregnancy discrimination issues, legal requirements and practical advice addressing student pregnancies and parenting (Husch Blackwell 2021). South African universities continue to report high rates of pregnancies, and the Department of Education is looking for solutions to address the crisis. Baloyi et al. (2021) campaigned for public awareness, parental involvement and community involvement. In South Africa, the policy that supports pregnant and parenting learners is called the Policy on Prevention and Management of Learner Pregnancy in Schools; however, this policy does not apply to tertiary institutions (Department of Basic Education 2021). This highlights a gap in the lack of policy development focusing on pregnant students in tertiary institutions. The learner pregnancy policy focuses more on learners in high school than students in tertiary institutions. The policy permits every pregnant person and teenage girl the right to free medical care from all public clinics and hospitals. The policy also allows pregnant female learners to continue with their studies, like their male counterparts who impregnated them (Department of Basic Education 2021). The other important piece of legislation in South Africa that protects the rights of pregnant and parenting students is the Women Empowerment and Gender Equality Bill 37005 of 2013 (South African Government 2013). This bill stipulates that women’s childrearing obligations should not lead to exclusion or dropout to gradually realise universal access to education. In addition, there must be an improvement in access to information on reproductive rights for women, especially young women (South African Government 2013). Although this policy advocates for the rights of pregnant and parenting students, the implementation thereof is not visible at the tertiary institution. Therefore, this study aims to explore the experiences of pregnant and parenting students at a tertiary institution in Gauteng province, South Africa.

## Research methods and design

### Design

This study followed the exploratory, descriptive and qualitative design (Brink, Van der Walt & Van Rensburg 2018). To encourage participants to share their experiences of being pregnant in the context of an academic setting, an exploratory, descriptive and qualitative research design was adopted.

### Setting

The research study took place at a university in Gauteng province, South Africa. The institution is located within a township of about 37 km, northwest of Tshwane, in Gauteng province.

### Population and sampling

The population for this study was pregnant and parenting university students. The target population was registered pregnant and parenting undergraduate university students at a university in Gauteng province. Undergraduate registered students who were pregnant, have had a pregnancy or were parenting and lactating during their tertiary education, and all undergraduate female students, 18 years and above were included in the study. All female students with no history of being pregnant were excluded from the study. In this study, the non-probability purposive sampling approach was employed. The researcher’s assessment of the participants’ experiences and familiarity with the study’s phenomenon informed the sampling strategy. The researcher selected the participants purposively because she saw them as being knowledgeable about pregnancy and parenting while in university. They were knowledgeable and had experience with the topic of interest because some of them were pregnant while others were parenting while they were students at the university. Fifteen participants made up the study’s sample size. Therefore, the researcher calculated the sample size based on the total number of individuals questioned at saturation (Holloway & Galvin 2017).

### Data collection

Permission to conduct the study was granted by the heads of departments as the gatekeepers before the researcher contacted the participants. The researcher contacted the participants to arrange for a date to conduct the interviews. Data were gathered through semi-structured interviews (Brink et al. 2018). A semi-structured interview guide was used, which contained research questions that addressed the demographic information and interview topics. The age range, educational attainment, number of pregnancies and marital status were among the demographic information. Thirty to forty-five minutes were allotted for the interviews. The venue was a suitable, peaceful setting that guaranteed comfort and seclusion. To prevent bias, the researcher employed a research moderator to oversee the interviews with subjects with which the students were acquainted. The moderator was an experienced qualitative researcher who had signed a confidentiality agreement. After participants gave their consent, the interviews were recorded using an audio tape recorder. The English language was used for conducting the interviews because it is the mode of instruction in the university, so all participants were conversant with the language. The central question followed by probing read as follows: ‘*What are your experiences regarding pregnancy and parenting as a student?*’

### Data analysis

Thematic analysis was used to analyse the data (Polit & Beck 2020). Full transcripts of the participants’ actual speech and facial expressions were then recorded. The researcher started by being familiar with the data by reading it multiple times to get a better understanding of the information. Then the data were coded by identifying emerging meanings and labeling to enable the grouping of similar codes. This was performed by dividing the data into more manageable chunks to facilitate the coding process. The codes were then assessed for emerging patterns and meaning, which led to the development of the themes. Similar codes with the same meaning were grouped to make themes. The emerging themes were then reviewed to check if they correlated with the data, and could be supported with the data. After determining the final list of the themes, the researcher gave them distinct names, which had an overarching meaning to the codes that were grouped. The transcripts were sent to an external coder to further analyse the data. The external coder helped with an independent analysis of the data, which led to a consensus meeting with the researcher to examine similarities and differences in the analysis by the researcher and the external coder. Patterns of similarities and differences in the data were found during the meeting. Both the researcher and external coder reached an agreement on the final themes and categories.

### Trustworthiness

Trustworthiness is a determination that a qualitative study is rigorous and of high quality, and the study is sound and adequate (Holloway & Galvin 2017). The authors made judgements of trustworthiness through the criteria of dependability, credibility, transferability, confirmability and authenticity. Prolonged engagement ensured credibility because the researcher was familiar with the participants because of her position as a lecturer at the chosen university. The researcher used transcriptions from recordings, observations of participants during interviews and field notes to ensure data triangulation. To eliminate bias, the researcher used a research moderator with experience in qualitative research, and all interviews were performed in the presence of the moderator. The researcher asked open-ended questions and interviewed each participant to fully understand their perspectives on the subject of interest. The sample size was determined using data saturation after the researcher conducted in-depth interviews that allowed for clarification of responses.

The authenticity of the data was guaranteed via recording and verbatim transcribing of interviews. When the researcher assumed a self-critical stance, she was motivated to focus on what was true about the study and avoid using her own thoughts and sentiments towards participants. The study’s dependability was verified via an audit trail that included the protocol, recorded interviews, full transcriptions and field notes. To establish confirmability, the researcher ensured that the data obtained were a true reflection of the experiences shared by the participants. Authenticity was assured by treating the information provided by the participants with balance and equity. The participants’ voices were reflected in the verbatim quotes. Throughout the interviews, member checking was performed. At the start of the session, the researcher provided the participants with an overview of their interview and continued to watch their reactions throughout the interview. The researcher saw member checking as a crucial technique in light of the study’s sensitivity.

### Ethical considerations

The Sefako Makgatho Health Sciences University Research Ethics Committee gave its clearance for the study (reference no.: SMUREC/H/57/2018). In addition, approval was received from the university’s Deans of the schools and heads of the departments. By giving participants the information, they needed to make an informed decision about participating or not in the study, the right to self-determination was upheld in this investigation. Participants were made aware that participants might leave the study at any time. By explaining the study’s methodology to participants and getting their informed agreement, the researcher avoided using coercion. Before they participated in the study, all study participants signed a consent form. To maintain participants’ confidentiality. The researcher in this study did not associate the data gathered from them with their names.

## Results

The participants were registered pregnant and parenting undergraduate university students at a university in Gauteng province. All participants involved were female black students who were either pregnant or parenting while studying at the tertiary institution. These participants came from the following Schools within the tertiary institution: the School of Medicine, School of Oral Health, School of Health Care Sciences, School of Science and Technology, and School of Pharmacy. The student participants were aged between 21 years and 30 years. The following themes emerged from the data analysis, namely: Theme 1: Emotional experience during pregnancy; Theme 2: Academic challenges during parenting; Theme 3: Experiences during antenatal care and Theme 4: Students’ resilience during pregnancy and parenting. The findings were presented as themes, and the participants’ verbatim quotes will be reflected to support the narratives about the themes.

### Theme 1: Emotional experience during pregnancy

The participants revealed that they experienced emotional turmoil during pregnancy, especially when they found out about the pregnancy. The emotional reactions were expressed through shock and fear upon realising that they were pregnant and in a bad situation. One of the participants reflected that the situation became really bad when she could not get support from her parents. The following are some of the voices of the participants:

‘For the first time after you discover that you are pregnant, you just shut down, you don’t care much about what are you writing tomorrow or what is happening.’ (Student participant 6, 26 year old, Female)

‘When I found out I was with my partner I asked him to be there with me because I did not want to digest this thing alone, but I was very scared though, It took me almost a month before I could tell my mother.’ (Student participant 2, 24 year old, Female)

‘It is really bad especially if you tell the news to your parents with the hope that they will comfort you and they don’t, It becomes really bad.’ (Student participant 1, 22 year old, Female)

The participant also revealed that pregnancy-related stress forced them to miss deadlines for submitting assignments and the pressure of having to end the academic year abruptly without being allowed to write the final examination. Some were even advised by lecturers to leave and abandon their studies because they had just given birth. One of the participants stated the following:

‘At that time I didn’t do anything because there were assignments that I didn’t even submit because of the stress, I was told that I should leave immediately because I am a parent now, and I was saying to myself “How do I leave because I’m almost done, what next?.’ (Student participant 5, 24 year old, Female)

The findings suggest that the students were unable to come to terms with the reality of being pregnant and being students at the same time. They were also expected to meet the requirements of their studies including submission of assignments on time. The other concern verbalised is the frustration of breaking the news to their parents who are not prepared to support them during this difficult time. The findings also reveal the emotional turmoil that is caused by the unplanned pregnancy that affected the student’s academic progress and the possibility of ending the academic year without writing the final examination.

### Theme 2: Academic challenges during parenting

Participants indicated that they experienced academic challenges after giving birth because they could not cope with their school work and being parents. They acknowledged their inability to balance school work and parenting, which necessitated frequent trips to home to care for the infant. It was challenging for some students to study at night, and during the day the baby is awake and needs her attention. Their caregivers or parents assisted with child care but wanted to be relieved over the weekends, which the parenting students usually use for studying. They stated the following:

‘My challenge was not throughout my pregnancy, my challenge is now after giving birth, that is where I have hit rock bottom when it comes to my school work, I had to go home every two weeks and it caused a huge imbalance when it comes to my school work.’ (Student participant 4, 26 year old, Female)

‘I could not study over the weekend because I cannot study at night. I prefer to focus on my schoolwork during the day because at night I’m not productive. During the day when I want to study, I can’t do anything if the baby is awake (Student crying).’ (Student participant 2, 24 year old, Female)

The participant narrated the frustration of not coping with schoolwork because of being a parent faced with two conflicting roles of being parents and students at the same time. This led them to change their study routines to accommodate both responsibilities. Some students went to the extent of studying in the early hours of the morning. While studying they were often interrupted by their babies who woke up for nappy changing or feeding. The drastic change in studying routine affected their academic performance because of missing class. The following are some of the expressions:

‘Studying has changed because I used to study late maybe from 1600 in the afternoon, when the lectures are over. I was studying up until late but now it has changed. When I get there I have to maybe bath him, and make sure that he sleeps and then I also sleep and wake up around 12 and study throughout until the early hours of the morning.’ (Student participant 12, 30 year old, Female)

‘I have changed my study programme, I don’t think this is working for me because I am not performing like I used to but I am passing, but the performance is not like before, I think it’s because the time is limited, what I do now is, I sacrifice attending classes, I no longer attend classes.’ (Student participant 7, 25 year old, Female)

### Theme 3: Experiences during antenatal care

The participants expressed frustration for being directed to the academic hospital for antenatal care (ANC) services, where they would have to stand in queues for hours before receiving assistance. They also expressed that during their ANC visits, it was difficult to ask pregnancy-related questions because nurses were unable to give them clear answers. Some students even questioned the credentials of some of the nurses assigned in the ANC Ward because they were just referred to the hospital without explanations. This was difficult because the students lacked knowledge and experience about pregnancy. The following are the participants’ expressions:

‘They will tell you that they are not dealing with pregnancy there and they will give you a letter and they tell you to go to the academic hospital, that is where you will receive help…Yes, the queue is just too much and is time-consuming.’ (Student participant 7, 25 year old, Female)

‘[*E*]specially if it is your first pregnancy you know nothing and when you go to ask they tell you, you must go to the hospital PHC, that is where they deal with pregnant people.’ (Student participant 8, 23 year old, Female)

The participants expressed that they frequently felt abandoned by the university because it did not offer essential medical services such as the antenatal clinic on campus. They also echoed the feeling of paying enough tuition to cover the antenatal care clinic on-site so that they can feel supported by the university during pregnancy. Furthermore, they suggested that they need to offer ANC services on campus, which will make them feel considered as women, not as children. The following are some of their expressions:

‘We are paying enough and they should have antenatal facilities for students. They can have that facility so that I can feel a little bit of support if I am pregnant.’ (Student participant 4, 26 year old, Female)

‘I don’t have to run away from school to get help. I can just get it within the facility, And they will feel like they are considerate towards us as women, because at the end of the day, we are women and no longer children. If they can get that antenatal clinic it can help a lot of students.’ (Student participant 6, 26 years old, Female)

The above finding reflects the need for essential medical services such as ANC services on campus. This will prevent students from consulting elsewhere outside the campus, which will be convenient for pregnant students.

### Theme 4: Students resilience during pregnancy and parenting

Despite the difficulties associated with pregnancy and parenting, some of the participants reflected resilience in coping with their situations. The participants expressed that they did not experience much of the negative effects of pregnancy because they had to ‘suck it up’, and displayed some maturity and an understanding of their situation. They revealed that they did not expect any special treatment during pregnancy and that they had to accept the situation as it was despite being exposed to triggering situations. Despite experiencing challenges, they were able to navigate through the process and did not demand any special consideration from their colleagues. They reflected the following:

‘When I was pregnant I did not experience much, I had this thing in my mind that there will be no special treatment that will be given to pregnant students, so I had to suck it up, you do not have special treatment.’ (Student participant 3, 25 year old, Female)

‘I went to some hospital kitchen and it was during my early pregnancy with morning sicknesses, the smell of the food, so I just had to suck it up and go through it. But it was not really that challenging if I may say, I didn’t become a cry-baby.’ (Student participant 3, 28 year old, Female)

The finding implies that some of the students had the will to cope with their situations. They showed resilience in dealing with their pregnancy and managed to deal with and cope with any challenges they were confronted with. Participants displayed a positive mindset to cope with the pregnancy and meet their academic requirements amid challenges.

## Discussion

The purpose of the study was to explore the experiences of pregnant and parenting students at a university in Gauteng province. The findings of the study suggest that despite students experiencing emotional challenges during pregnancy and parenting, the university did not provide emotional and academic support. Pregnant and parenting students wanted to be treated as mature, responsible and self-directed adults, so they needed ANC services to be available on campus. Participants also suggested that they need to be formally supported during pregnancy and parenting so that they can be able to cope with their academic workload. In addition, participants want support from their parents to offer emotional and physical support during pregnancy and parenting. However, Phiri et al. (2023) asserted that when a female student becomes pregnant, she says farewell to youth and adds the dignity of motherhood to her academic work.

Obligations of parenthood and student life are frequently irreconcilable. This is similar to what the current study found, which reflected that students could not cope with their academic responsibilities and being parents. Owoko (2017) agreed that it can be difficult to study when expecting a child and raising a family. This study’s findings found that some students were informed to leave their studies as soon as they gave birth because they had to deal with their newborn babies. This reflects the inconsistencies in managing pregnant and parenting students at universities because of a lack of commitment from university staff and management. Instead of advising parenting students to leave the university, some studies suggest that students can still be accommodated if the academic plan is adjusted to suit their needs. This was recommended by Thabethe et al. (2020) who emphasised support for developing the lesson plan with pregnant students who identified themselves to develop a graduation plan that is specific to each student’s needs. Moreover, tertiary institutions should offer pregnancy and parenting students individualised support programmes. Studies by Haufiku (2023) and Phiri et al. (2023) highlighted the need for compliance initiatives regarding issues involving student assistance for parenthood and pregnancy. Accommodating parenting students in universities may ease some parenting issues, such as remaining closer to educational institutions and refraining from bringing children to parents who live extremely far from educational institutions.

Ajayi et al. (2023) indicated that most campuses lack a framework for identifying student parents and this gap can be closed through outreach efforts that can identify student parents as early as possible. This is consistent with this study because students reflected that they felt abandoned by their institution because they did not have access to essential healthcare services such as antenatal care to cater for pregnant students. Another gap identified by Thabethe et al. (2020) is the unavailability of childcare facilities for students, which leads to students worrying about caring for their children while at university. This was supported by Haufiku (2023) who stated that amenities such as on-campus child healthcare centres or the availability of support funds to help students pay for off-site daycare should be made available.

Families, partners, tertiary institutions and peers make up pregnant students’ mesosystems (Jochim et al. 2021). Their supportive efforts towards pregnant and parenting students are important in assisting with their academic progress. The students in this study revealed that they lacked support during this difficult time, especially from their parents. Pregnant and parenting students can focus on their schoolwork if they receive financial assistance following the birth of their child to help with basic needs (Assini-Meytin, Mitchel & Lewis 2018). Young mothers who receive support experience less stress and burden and this fosters emotional connections, resilience and empowerment (Dhaka & Musese 2019). In contrast, this study reflects that the university environment is not supporting pregnant and parenting students. This is consistent with a study by Kiburwi (2018) who reiterated that students who fall pregnant and raise children while at university should not anticipate receiving assistance from tertiary institutions. This means that despite institutions allowing students to fall pregnant and continue with their classes, there is no concern for a conducive environment for them to complete their studies. For instance, university bylaws in countries such as Tanzania do not permit female students who are pregnant or nursing their infants to reside in the students’ residences, even breastfeeding is not permitted on campus (Kiburwi 2018).

Pregnancy among tertiary students in South Africa and other countries is still a public health and educational issue that calls for the cooperative engagement of key stakeholders, including lecturers (Matlala 2017). Furthermore, Conradie (2019) hinted that the environmental, academic and social factors in universities should play a larger role in providing support to pregnant and parenting students. If pregnant and parenting students receive well-coordinated academic and social support, the institutions’ commitment to their future will be applied with fluency, resulting in the completion of education (Conradie 2019). However, Stephens (2017) and Phiri et al. (2023) observed that not all students’ pregnancies have a detrimental effect on their academic achievement because some complete their education without too many challenges. Conversely, pregnancy in the absence of appropriate support has an impact on pregnant students’ academic performance (Nkosi, Makhene & Matlala 2019).

## Conclusion

The purpose of the study was accomplished by exploring the experiences of pregnant and parenting students at a university in Gauteng province. In conclusion, this study suggests that pregnant and parenting students do not receive adequate academic, emotional or social support from parents, the university and other relevant stakeholders during their period of study. As a result of the competing and incompatible responsibilities of parenting and being students, pregnant and parenting students face difficulties. These difficulties have an impact on academic advancement and also cause certain lecturers and students to make disparaging remarks towards pregnant and parenting students (Jochim et al. 2021). Moreover, some lecturers, in addition to family and friends, did not provide emotional and social support to pregnant and parenting students in this study. For pregnant students to gain from social assistance in handling their studies, lecturers and relevant stakeholders such as psychologists and social workers must participate in the process. This study highlighted a gap in the literature regarding formal support to be offered to pregnant and parenting students in universities.

This study only focused on undergraduate students from tertiary institutions who were pregnant or parenting their infants. Therefore, the findings do not reflect the experiences of post-graduate students from tertiary institutions. It is recommended that students in tertiary institutions should be offered a well-established catch-up programme as part of their curriculum to support pregnant and parenting students. All pertinent parties involved in facilitating learning in tertiary institutions should be encouraged to participate in induction and in-service education programmes so they may become knowledgeable about how to support pregnant and parenting students. Furthermore, the policy intended to assist student mothers should be clear to both the academic staff and students.
